# Coaching Robots for Younger Seniors: Do They Get What They Expect? Insights from an Austrian Study

**DOI:** 10.3390/ijerph20042965

**Published:** 2023-02-08

**Authors:** Cornelia Schneider, Hafsa Bousbiat

**Affiliations:** Institute of Computer Science, University of Applied Sciences Wiener Neustadt, 2700 Wiener Neustadt, Austria

**Keywords:** Ambient Assisted Living, AAL, social robot, usability, user experience

## Abstract

To support the increasing number of older people, new (assistive) technologies are constantly being developed. For these technologies to be used successfully, future users need to be trained. Due to demographic change, this will become difficult in the future, as the resources for training will no longer be available. In this respect, coaching robots could have great potential to support younger seniors in particular. However, there is little evidence in the literature about the perceptions and potential impact of this technology on the well-being of older people. This paper provides insights into the use of a robot coach (robo-coach) to train younger seniors in the use of a new technology. The study was carried out in Austria in autumn 2020, involving 34 participants equally distributed among employees in their last three years of service and retirees in their first three years of retirement (23 female; 11 male). The aim was to assess participants’ expectations and perceptions by examining the perceived ease of use and user experience of the robot in providing assistance during a learning session. The findings reveal a positive impression of the participants and promising results for using the robot as a coaching assistant in daily tasks.

## 1. Introduction

In recent years, against the background of demographic change, a variety of technologies and services for older people have been developed in various projects [1,2,3], so-called Ambient or Active Assisted Living systems (AAL for short). AAL is an umbrella term for a variety of systems ranging from wearable devices and cameras to non-invasive solutions [4,5] that promote quality aging in the digital age. However, it has become apparent that many of these technologies require training [6,7,8]. Due to the retirement of baby boomers (in Austria, the cohorts 1956 to 1969 [9]), the number of persons of working age is decreasing and the number of inactive persons is increasing [10]. The Elderly Dependency Ratio (EDR), a measure capturing the ratio of the number of old adults (over 65 years of age) to productive adults (between 15 and 64 years of age), has increased by 6.2% in EU Member States in the last decade [11] and is projected to reach 57% by 2100 [12], which is about twice the value calculated for 2021 (32.5%).

The working lives of the baby boomers were and are characterized by the information age [13]. Therefore, it can be assumed that they are more familiar with technology, unlike the generations before them. Statistics show that a growing number of them have been involved with technological systems, including social media and online resources, in recent years [14]. Particularly, the baby boomer generation would be able to use and benefit from new technologies that support them in aging if trained properly. In Germany and Austria, for example, around 90% of 55- to 64-year-olds use a smartphone [15,16]. This translates to the fact that more people may want to use new technologies in the future to support them in aging, but due to the demographic change, fewer people will be available to train them.

In this respect, social robots bear great potential to address this issue in the future. Social robots can be described as artificial agents with the characteristics of humans or animals [17]. They are currently already used in education, particularly in teaching technical skills, especially to children and young people [18]. With respect to older people, they have so far been used mainly in long-term care settings and for people with dementia, focusing on nursing homes and community dwellings [17,19]. More precisely, the use of coaching robots for older people is an active area of research with the goal of improving their quality of life and independence. In this regard, a study conducted in North America demonstrated that older adults have preferences for robot assistance when it comes to tasks related to chores, manipulating objects and information management [20]. In addition, recent research emphasizes the need to consider the fears and desires of target users before introducing social robots [21]. These studies highlight the importance of user perceptions when it comes to realizing the full potential of social robots and show that negative perceptions could be a barrier to the success of this technology. However, studies with real robots show good acceptance. An example of this is the study presented in [22], in which an assistive robot was used to aid seven patients with mild cognitive impairment over a period of 118 days. This has also been highlighted in similar studies evaluating the effect of social robots on older adults’ motivation to engage in physical exercises [23,24]. Another study evaluating the readiness of older adults to use social robots was presented in [25], highlighting that social robots are perceived as futuristic technology to be used in the future, but less likely in the present. This demonstrates an important gap between studies involving real robots and studies only reporting subjective opinions of older adults. It is not clear whether these perceptions/opinions have a direct effect on the potential of using social robots and no clear answers can be found in the literature about if these perceptions are affected by the fact that older adults are less familiar with robots. Therefore, there are several challenges and limitations that need to be considered when developing and implementing these robots.

One of the main challenges is to ensure that the robots are easy to use and can be understood by older people. This includes designing user-friendly interfaces and providing clear instructions and feedback [26,27]. Another challenge is adapting robots to the individual needs and preferences of older adults [27]. This could also include the development of personalized interfaces [26] that, for example, allow coaching robots to perceive learning progress or even adapt to the user’s learning pace. [20] Furthermore, there is a need for evaluating the effectiveness of the robots, both in terms of their technical capabilities [26,27] and their impact on the quality of life of older people. Overall, while the use of coaching robots for older people has the potential to improve their quality of life and independence, it is important to carefully consider these challenges and limitations when developing and implementing these robots. In addition, there is little evidence for the case of younger seniors and technology education/training. Moreover, in some cases, real robots were not used [28], making it difficult to assess the actual perception of seniors towards this technology and thus leading to limited conclusions.

The European AgeWell [29] project addressed, among other things, the question of whether social robots can help train younger seniors (the baby boomer generation) in new technologies in the future, thus ensuring their digital connectivity. This paper therefore investigates the general perception as well as the real interaction with a social coaching robot in the baby boomer generation (older employees and people just retired). In the present study, we therefore examined the use of a coaching robot in training sessions and described participants’ perceptions and experiences of interacting with the robot. Specifically, we observed participants’ expectations before using the robot and their perceived ease of use and experience after interacting with the robot. To achieve the goals of the study, we used a real robot coach (robo-coach) with software developed specifically to support the target group of participants.

The remainder of the paper is structured as follows: Section 2 introduces the robo-coach developed in the AgeWell project, Section 3 describes the study design and the different aspects investigated, including recruitment strategy and the methods for data collection, Section 4 presents the results, in Section 5 the results are discussed, and Section 6 provides a conclusion.

## 2. Project Background and Robo-Coach

The European AgeWell project [29] focused on developing an Embodied Conversational Agent (ECA) to support young seniors age actively and healthily. For this purpose, an ECA/virtual coach was developed in the form of an avatar running on a smartphone [30,31]. In addition, a robot was implemented for training purposes, including training in the use of the ECA. Subsequently, this paper deals with the use of the robot. The ECA itself is not the focus of this paper. More precisely, the robot acts as a coach for the participants in filling out a questionnaire or in explaining the project and the different components of the ECA through visual and auditory explanations.

### 2.1. The Robo-Coach

Even though future generations of younger seniors are increasingly familiar with technology, new applications often require training tailored to the target group [32,33], which is usually associated with considerable human resources and costs. This is where the AgeWell robot comes in, taking on the role of a digital training coach (robo-coach) in the project.

**Figure 1:**
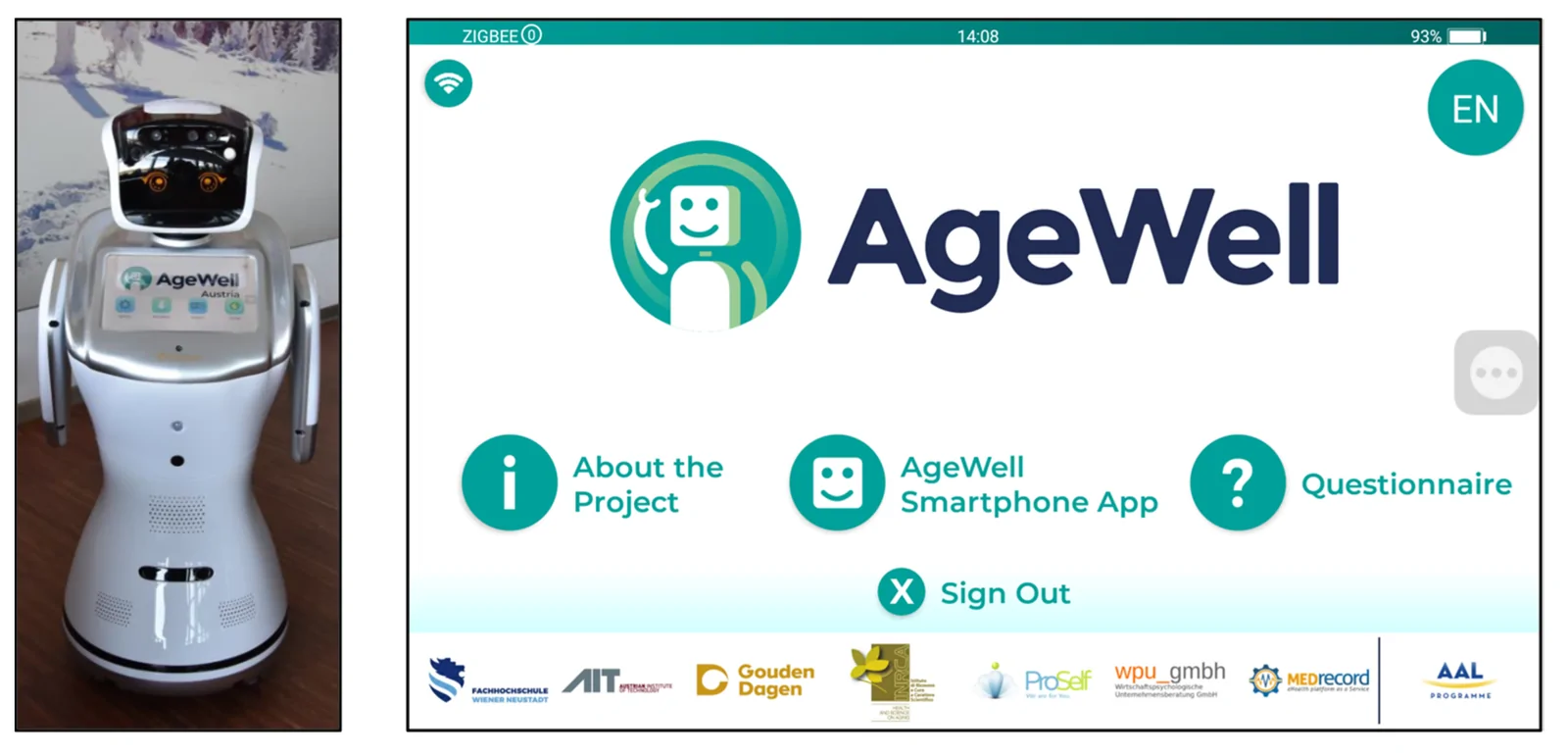
Sanbot Elf and main screen of the robo-coach.

Figure 1 shows the robo-coach and its main interface. The robot used is a Sanbot Elf [34], which was selected based on a previous study [35]. The robot was selected based on (i) use-specific criteria, (ii) technical and physical requirements, (iii) input and output capabilities, (iv) connectivity, (v) (further) development capabilities, (vi) cost, and (vii) market availability. The selected robot Sanbot Elf has a human-like appearance with advanced but limited communication capabilities. More specifically, the robot is able to explain and interact with humans both visually and verbally, but in a predefined context.

**Figure 2:**
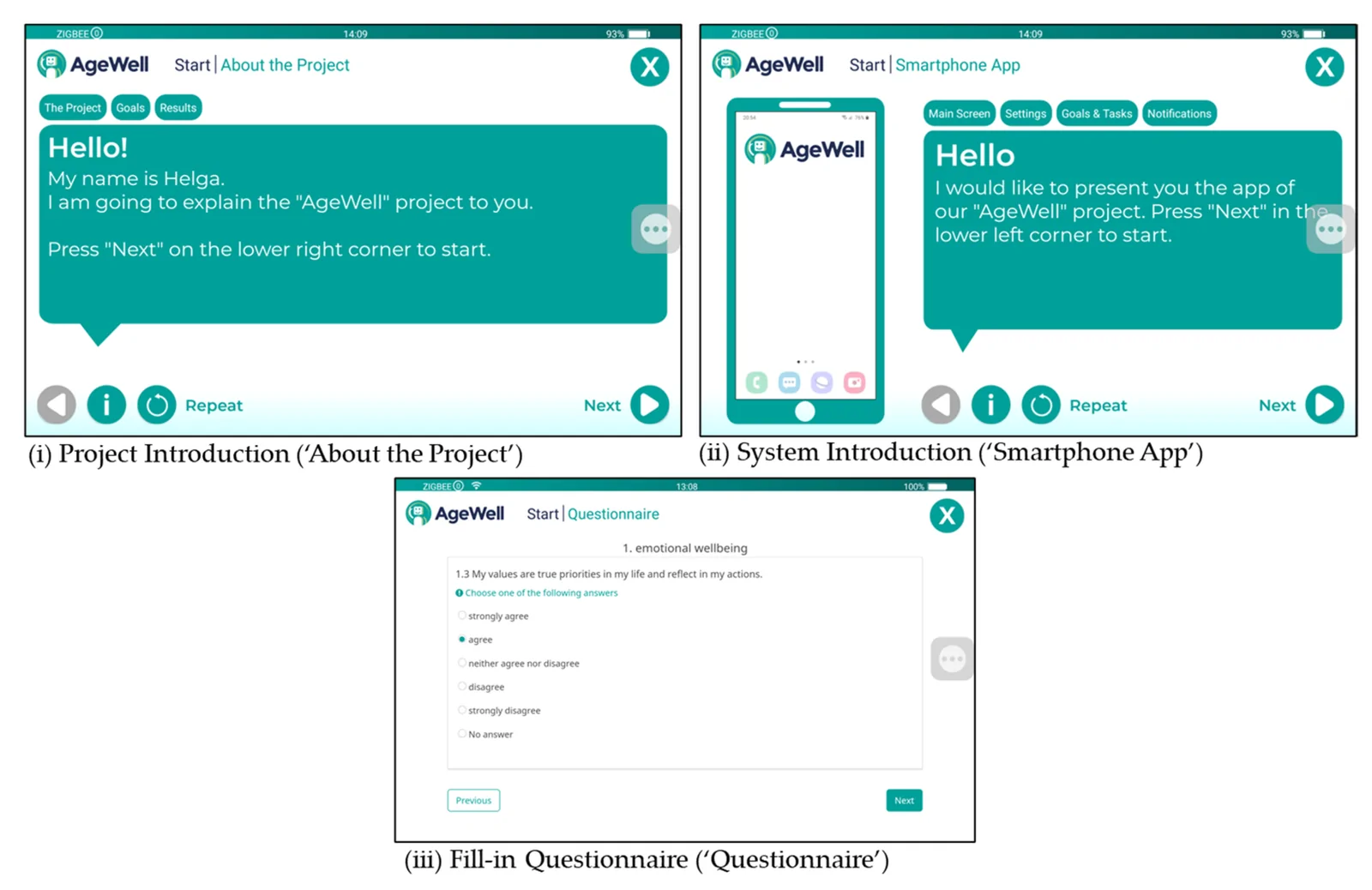
Interfaces for the three functions of the robo-coach.

The main goal of using a robot in the AgeWell project was to show, using the ECA as an example, that training younger seniors by a robo-coach is possible in order to have alternatives to training with humans in the future. For this, the robo-coach had to perform the following tasks (cf. Figure 1—main screen): (i) introduce the project and its goals to users (‘About the Project’), (ii) train users on the project’s smartphone app (‘AgeWell Smartphone App’), and (iii) have users complete a simple questionnaire (‘Questionnaire’) [35]. More details on the three main functions are shown in Figure 2:

(i) *Project Introduction (‘About the Project’)*: The aim of this function was for the robot to introduce the AgeWell project and its aims to the target group.
(ii) *System Introduction (‘AgeWell Smartphone App’)*: This function should support system training. The robot introduced the ECA to the target group. The aim was that only little or no additional system training was necessary after the robot’s system introduction.
(iii) *Fill-in Questionnaire (‘Questionnaire’)*: To use the ECA, a well-being questionnaire has to be filled out. The robot was used to support this process.

Apart from a login and the three functions presented, no other functions could be used on the robot, since the robot was operated in kiosk mode. Login with a QR-code was required to access the training. After logging in, the robot displayed a screen with a start button that provided access to a spoken explanation that guides participants through the three functions. While the users followed the robot’s explanations of the “system introduction” function, they also had access to the ECA application running on a smartphone to try out what was shown for themselves right away. In addition, the robot offered the possibility to repeat explanations if the user had not understood them. In the opposite case, the robot asked users to confirm their understanding before proceeding to explain another task. Additionally, users had also the opportunity to navigate between different explanations of the robot.

### 2.2. Brief Overview of the ECA App Used

**Figure 3:**
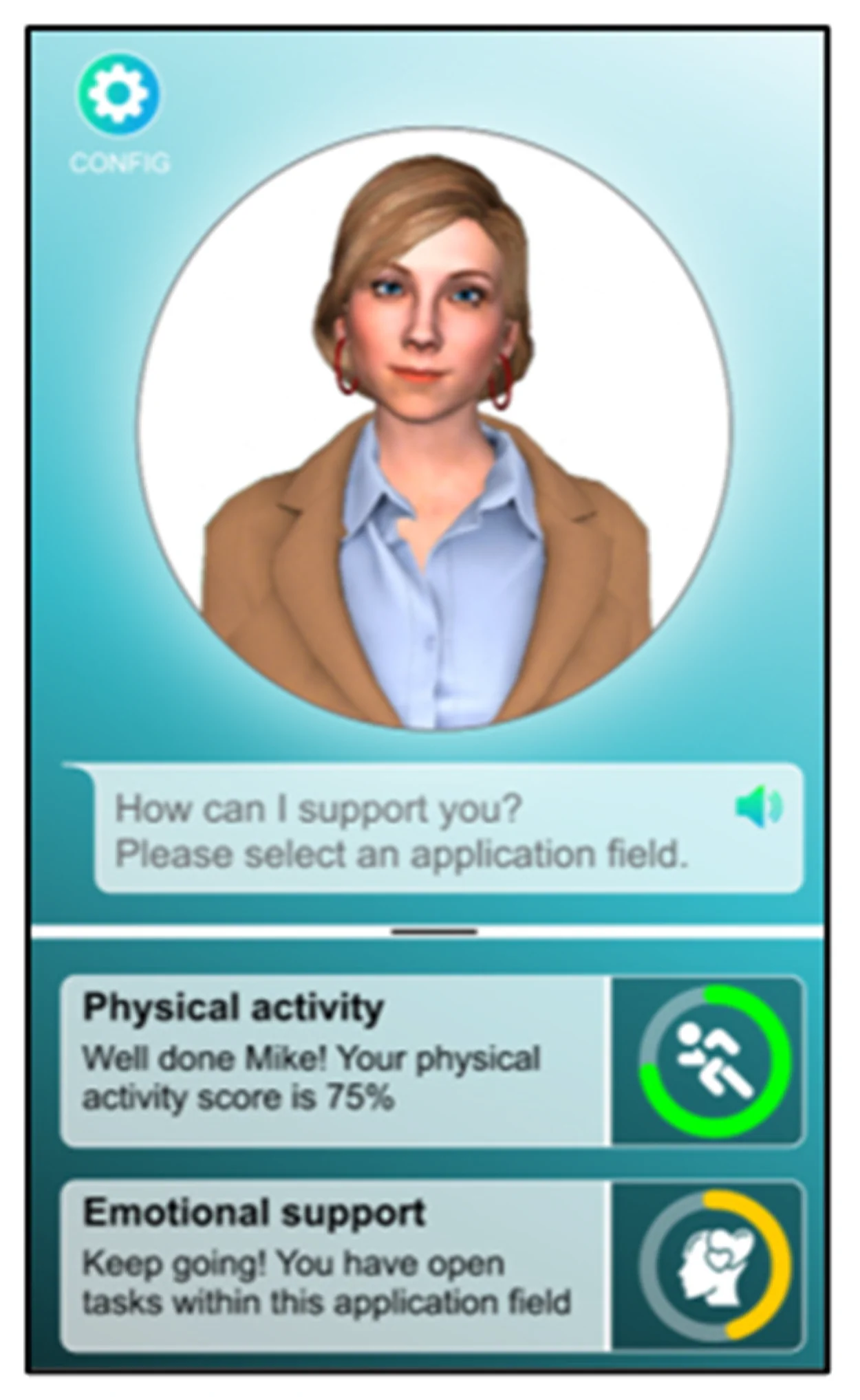
ECA application.

The developed ECA application, which the robot explained to the users in the “system introduction” function, is shown in Figure 3. The app consisted of an avatar that guided the user through the two functions: (i) physical activity and (ii) emotional support. The “Physical activity” function allowed activities such as walks to be scheduled for a specific duration on specific days of the week. The “Emotional support” function provided the opportunity to plan and conduct positive psychology exercises. The application was developed by a team of the Austrian Institute of Technology [36] and was used in our study as content to test the robot’s function “System Introduction”.

## 3. Methods

This paper aims to investigate the general perception as well as the real interaction with the robo-coach in the baby boomer generation. Thus, a study was conducted to examine the use of the robo-coach in training sessions to describe participants’ perceptions and experiences of interacting with the robo-coach. Particularly, the participants’ expectations before using the robo-coach and their perceived ease of use and experience after interacting with the robo-coach are examined.

### 3.1. Study Design

The study was conducted with 34 participants in Lower Austria in September 2020. The study (including the informed consents) was reviewed and approved by the independent ethics board of the University of Applied Sciences Wiener Neustadt (17/08/2020; chairperson: Nimmerichter Alfred).

We focused on white-collar workers in good health (no limitations in mobility or cognition) in transition to retirement (baby boomers). In the process, we targeted two target groups (i) white-collar workers who are currently employed but close to retirement age, i.e., 55 years or older (maximum three years before retirement), and (ii) white-collar workers who have just retired (in the last three years) and are 55 years or older. They also had to be interested in the project and be able to sign a consent form. Furthermore, the aim was to involve both groups (employed and retired) in equal numbers. Recruitment was carried out through business networks, clubs and personal contacts. As a COVID-19 wave was just building up in September 2020, measures were developed for the implementation of the study: (i) participants had to wear a mask, (ii) handshaking was avoided and (iii) all devices were disinfected after each participant.

### 3.2. Intervention

The intervention took place in a usability lab and was scheduled for 60 min per participant. Demographic data, affinity for technology, and expectations of participants with respect to the robot were collected prior to the intervention using a paper-pencil survey (Table 1). The intervention with the robot—‘Project Introduction’, ‘System Introduction’ and ‘Questionnaire’—lasted between 30 and 40 min. After the intervention, participants’ perceptions were examined (perceived ease of use and user experience of the robot) using a paper-pencil survey.

The intervention process can be summarized in six steps: (i) demographic data were collected by people accompanying the study, (ii) participants filled-in a pre-intervention survey concerning their expectations about the robot, (iii) participants received a short introduction into how to use the robot and the three functions by people accompanying the study, (iv) participants used the robot themselves without support (if support was needed, this was documented by people accompanying the study), (v) participants filled-in a post-intervention survey and (vi) people accompanying the study asked participants questions about the project and had them perform simple tasks with the ECA to see if the functions ‘Project Introduction’ and ‘System Introduction’ were successfully used.

At this point, we note that people accompanying the study in the final step of the intervention only took notes on the correct or incorrect performance of the tasks and did not conduct interviews.

### 3.3. Measures

To investigate the general perception as well as the real-life interaction of the study participants with the robo-coach, recognized measures and adaptations thereof were used. Table 1 gives an overview of the measures used in the four surveys. The most important aspects and adaptions of the measures are described in the following.

#### 3.3.1. Affinity for Technology (ATI) Scale

This scale assesses the user’s affinity for technology interaction. It is used, among other things, to characterize user diversity in system usability tests [17]. Although there are several scales available to measure technology affinity, we chose the ATI scale because, with only nine items, it provides a good balance between the factors of time, homogeneity, and clarity.

#### 3.3.2. System Usability Scale (SUS)

To quantify the usability as perceived by the participants, a system usability scale (SUS) was used. The SUS is composed of ten items on a five-point Likert scale [37,38,39]. To account for possible bias due to lack of attention when completing the questionnaire, the scale alternates between positive and negative items. The items in the scale represent a series of positive sentences that participants can strongly agree or strongly disagree with. For our study we used SUS at two different times, before and after the first interaction with the robot. Before the interaction, we used two adapted questions to obtain an idea of what participants think about support needs and learnability when they first see the robot:

- I think that I would need the support of a technical person to be able to use the robot. (Adapted question number 4 of the SUS)
- I think that I would need to learn a lot of things before I can get going with the robot. (Adapted question number 10 of the SUS)

After the first interaction with the robot, we used all ten items of SUS.

#### 3.3.3. User Experience Questionnaire UEQ Short Version

We planned the use of UEQ before and after the first interaction with the robot to measure the impact of the interaction on participants’ opinions. To keep within the planned time frame of 60 min for each study participant, we decided to use UEQ short (UEQ-S). It consists of only 8 items instead of 26 items using a 7-point Likert scale and focuses on measuring the two dimensions of pragmatic and hedonic quality [40,41,42].

#### 3.3.4. AttrakDiff (Adapted)

For measuring the attractiveness of the three functions of the robo-coach we used an adapted short version of the AttrakDiff questionnaire with six items [43,44] using a 7-point Likert scale. This questionnaire was used in the post-intervention survey.

#### 3.3.5. Tasks/Questions to Check if the Training Has Worked

To assess whether the participants understood the content of the ‘Project Introduction’, we asked three comprehension questions with predefined answer options (five each) and one open question. To check whether the ‘System Introduction’ works, we prepared nine tasks that the participants had to solve with the ECA after the training in front of the study accompanists.

## 4. Results

### 4.1. Description of the Sample

In total, 34 participants from Austria were involved in the study, including 23 women and 11 men (Table 2). The participants were distributed across two Austrian regions, mainly Vienna and Wiener Neustadt. They were also evenly distributed across two main employment categories: (i) 17 retired individuals and (ii) 17 employees nearing retirement. The average age of participants was 61, with an average age of 63 for retired individuals and 59 for individuals nearing retirement. All participants reported using a smartphone.

### 4.2. Technology Affinity of the Sample

**Figure 4:**
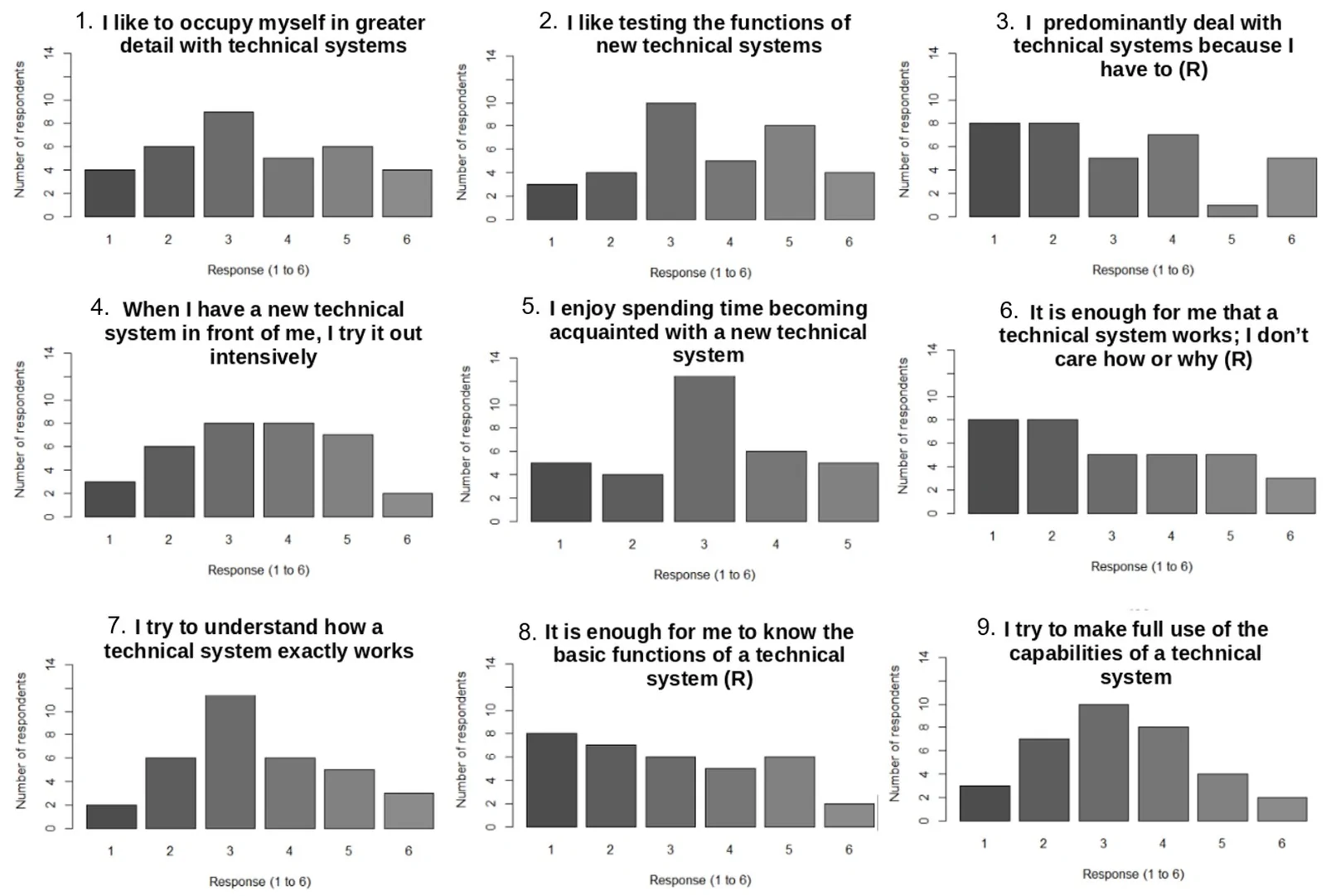
Results for different items of the ATI scale (*n* = 34; R = reverse coded).

Of the 23 female participants, 13 reported using a tablet, and of the males, 7 out of 11 (Table 3). Participants’ affinity for interacting with technology in general was assessed using the ATI scale. The Cronbach’s value for the entire group of participants was found to be 0.856, indicating a high internal consistency of the ATI scale for the group of participants under consideration. The bar charts for the items showing the frequency of agreement are shown in Figure 4 (no missing data).

Furthermore, the correlations between the items were all positive. The third and eighth item (reversed) had the lowest correlations with other items, with values between 0.01 and 0.63 and between 0.09 and 0.49, respectively. Other items correlated with each other within a range of 0.32 to 0.91. A mean value of 3.26 was obtained for the scale (Table 4). This score was below the average ATI score for the whole population, which is 3.5 [45]. Nonetheless, considering the target group (older employees in good health and retired older people in good health), a value of 3.26 remains acceptable. Based on the standard deviation and the minimum and maximum values, it is also clear that the individual results vary greatly between the participants. Specifically, 11 participants had an ATI score above 3.5, and 23 were slightly below average. When results are broken down by participants’ educational level, it appears that participants with higher levels of education (i.e., A-levels or university education) had lower ATI scores than participants with lower levels of education. One explanation for these results could be related to the age of the participants in each group. As shown in Table 4, participants with higher levels of education were older than participants in the other group. In addition, individuals with higher levels of education are often more critical, which may have influenced their responses. In summary, the results so far show that while participants are familiar with the use of common digital tools, they are skeptical of new technologies such as robots. This aspect was particularly evident in the third, sixth and eighth items of the ATI scale. These three items measure the overall level of curiosity that the participants have towards using a technical system. They are marked in Figure 3 with the letter (R) to indicate that these items were reverse coded as specified. For these questions, the participants’ response tended to be negative. In summary, the majority of participants show a low level of curiosity about technical systems. They believe that it is sufficient to know the basic functions and prefer to use them only when necessary.

### 4.3. Expectations of the Participants before the Use of the Robot

**Figure 5:**
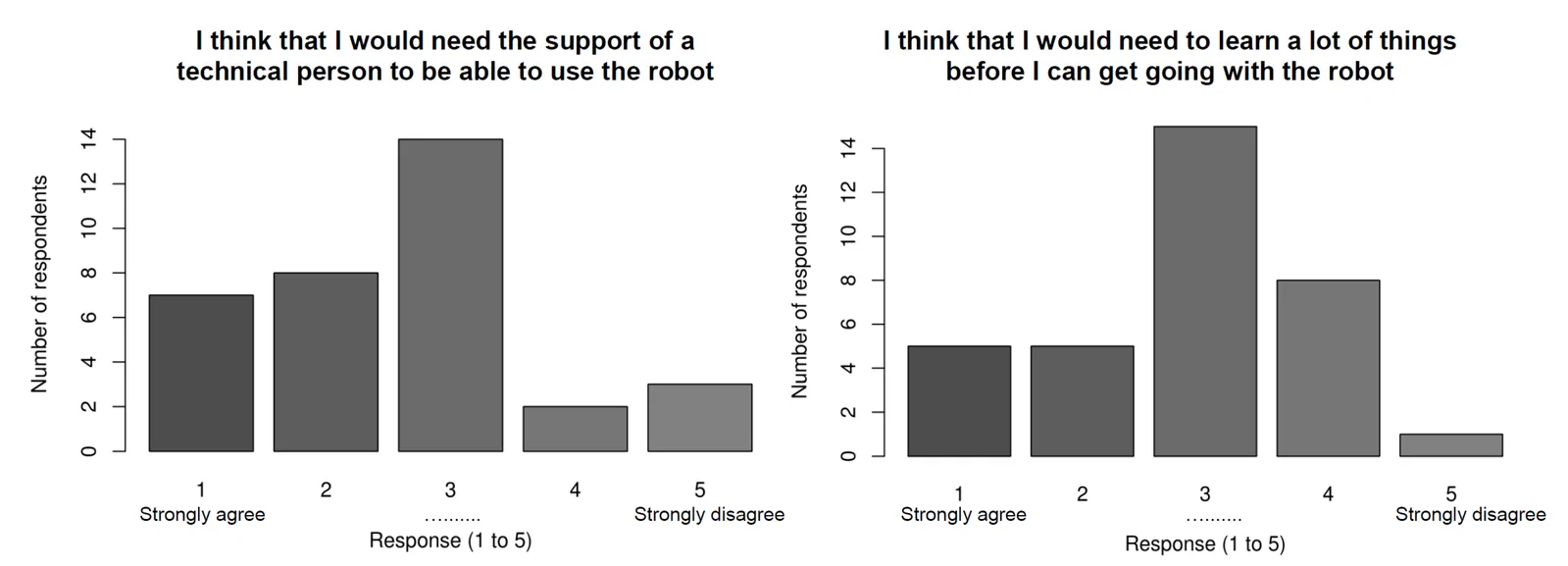
Results for the SUS items (*n* = 34).

Participants’ expectations with respect to support needs and learnability were elicited prior to interaction with the coaching robot through two adapted SUS questions (Section 3.3.2) and the UEQ-S (Section 3.3.3) to determine participants’ pragmatic and hedonic expectations. Expectations for support needs and learnability are shown in Figure 5. The results show that 45%, or 15 participants, indicated before using the robot that they would need the assistance of a person with technical skills to use the robot. Another 40% were not sure whether they would need assistance or not. These results show that only 15% of the participants were confident that they would be able to operate the robot without assistance, of which only two participants had a high ATI score (>3.5). On the other hand, about 55% of the participants were about equally in favour and against the need for extensive training before starting with the robot. In addition, about 45% were not sure if they would need much learning, with most of them having a high ATI scale (>3.5).

As shown in Table 5, participants rated the pragmatic quality of the robot as slightly positive/neutral before they even used it, while the hedonic quality was rated very positively. Here, values between −0.8 and 0.8 represent a neutral evaluation of the corresponding scale, values >0.8 represent a positive evaluation, and values < −0.8 represent a negative evaluation [46]. All subitems of hedonic quality were rated positively. Two items of the pragmatic quality were rated positively and two neutral.

In summary, the expectations of the participants, as measured by the two adapted SUS questions and the UEQ-S, can be summarised as follows: (i) Participants highly anticipated that they would need the assistance of a third person before using the robot, indicating low confidence in their ability to operate the robot alone. (ii) Participants had neutral/slightly positive expectations of the robot’s practicality and functionality, as measured by the sub-items of pragmatic quality. In contrast, they rated hedonic quality very positively, evaluating non-task-oriented aspects of the robot.

### 4.4. Participants’ Perception and Assessment after Using the Robot

After testing the robot, the SUS, UEQ-S, and an adapted version of the AttrakDiff questionnaire (for each of the three functions of the robot) were conducted. Participants rated both the pragmatic and hedonic qualities of the robot positively after using it. In contrast to participants’ expectations regarding the complexity of the robot, they also found it easy to use. Ratings of other sub-items of pragmatic quality also increased. The hedonic quality decreased slightly. Nevertheless, the results remain positive. Table 5 also shows the results of a t-test to test the differences between the responses before and after the use of the robot for the individual items of the UEQ-S. As Table 5 shows, only two items of pragmatic quality (ease and clarity of the robot) showed significant differences before and after using it. Participants underestimated ease and clarity before using the robot.

**Figure 6:**
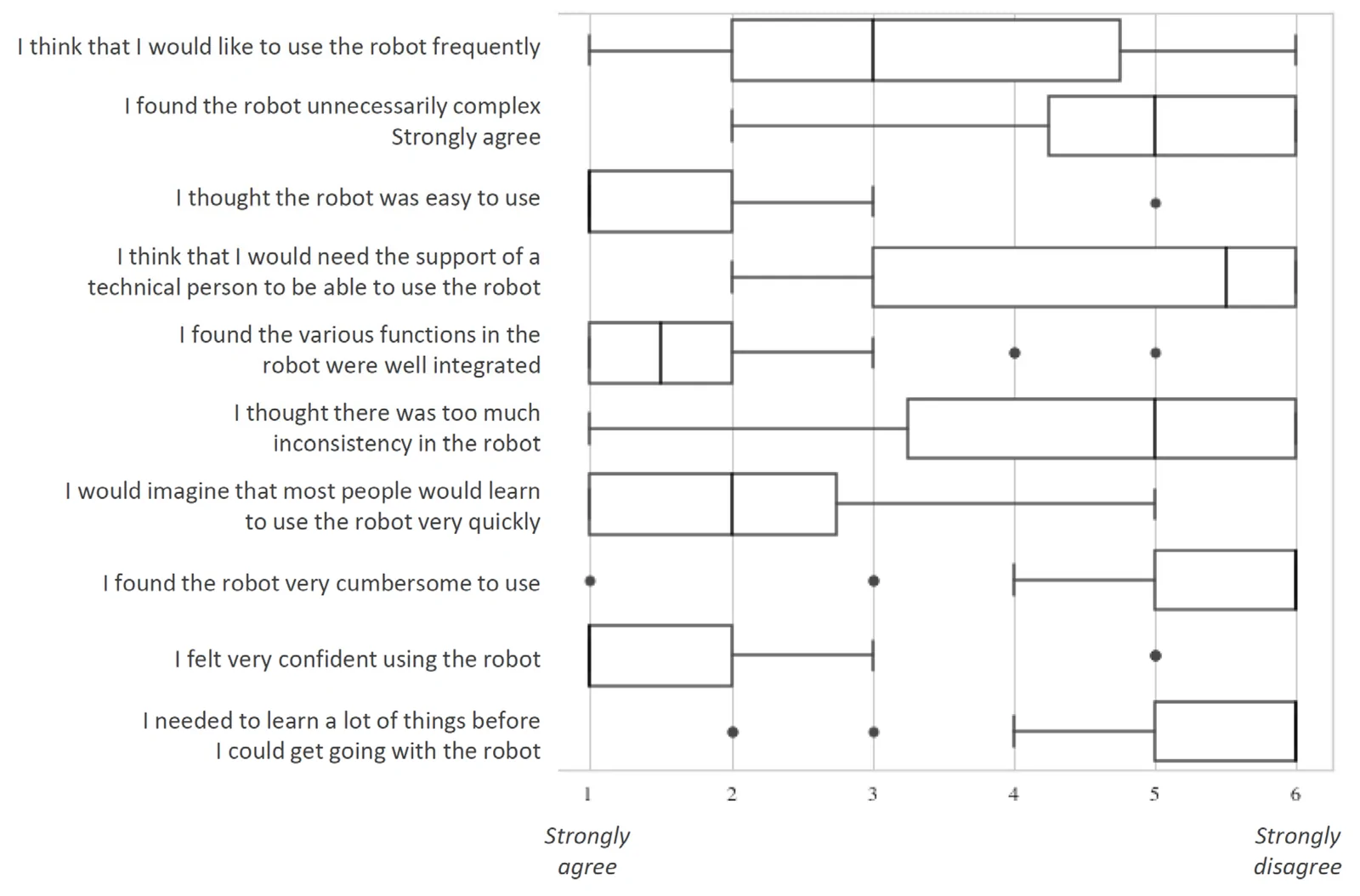
Results for system usability scale after using the robot (*n* = 34).

Figure 6 illustrates the results of measuring the usability of the system using SUS. As the figure shows, the participants showed a very positive attitude towards the robot. For the two items measured before the robot was used, it was interesting to note that participants drastically changed their perceptions after using the robot. They were neutral or did not see the need for support in handling the robot. In addition, there was no longer a perceived need for an extensive learning process to become familiar with the robot. Furthermore, the other items suggest that participants’ interaction with the robot changed their perceptions regarding the ease of using the robot and their confidence in their ability to use the robot. A summary of participants’ SUS scale responses based on education level is shown in Table 6. It shows that participants with higher levels of education had lower SUS scores. Similarly to the ATI scale, lower values are also observed for participants with higher levels of education.

**Figure 7:**
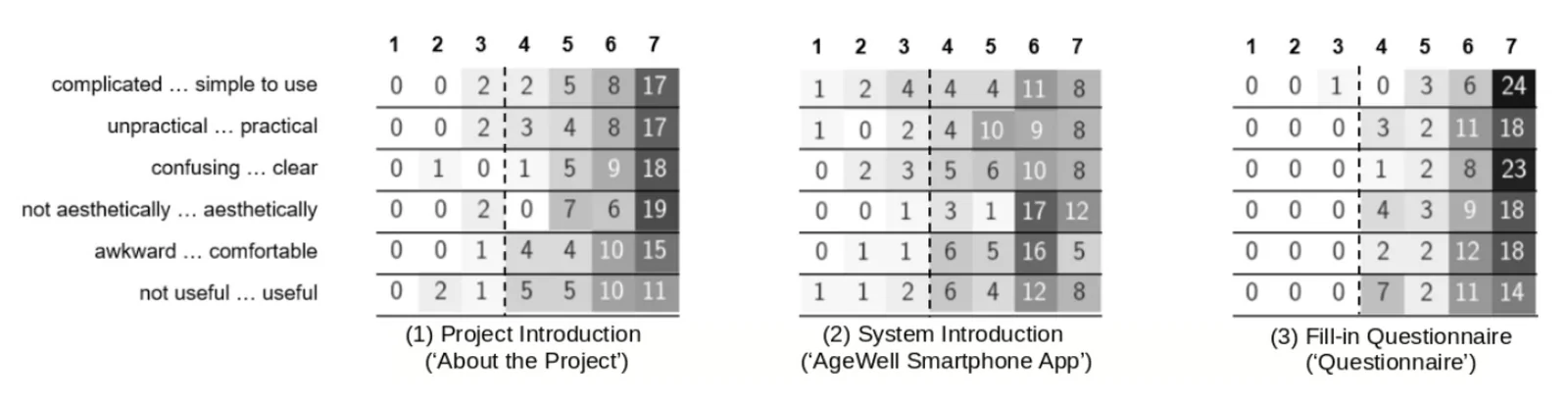
Attractiveness of the three functions of the robot (*n* = 34).

The results of the participants’ evaluation of the attractiveness of the three functions of the robot are shown in Figure 7. It shows the number of participants’ responses for each item of the questionnaire, measured on a seven-point Likert scale, with higher values indicating a positive evaluation of the different functions of the robot. As the figure shows, the majority of participants gave neutral to positive ratings to the various functions of the robot. The results for the Fill-in Questionnaire (‘Questionnaire’) function were particularly striking in this regard, as all but one participant gave a positive rating, with simplicity and clarity receiving the highest positive rating. The Project Introduction (‘About the Project’) was rated somewhat lower. Nevertheless, the overall evaluation remains consistently positive (only three participants did not find it very useful). Since the participants received at least a brief explanation of the project before using the robot, this was to be expected. On the other hand, a larger number of subjects rated the introduction to the System Introduction (‘AgeWell Smartphone App’) negatively. This could be related to the complexity of the ECA rather than the robot’s function.

Tasks that had to be completed after the ‘System Introduction’ also indicate this. As can be seen in Table 7, more than half of the participants had problems deleting a task in the smartphone app (ECA). In addition, a quarter of the participants failed to add an activity using the app. Although it is difficult to find a precise explanation for this, we suspect that they may be related to the design and implementation of the two tasks in the mobile application. The process of deleting or adding a task involved several steps that may have made it difficult for participants to remember and reproduce the task when they performed it again. Furthermore, one-eighth could not complete an activity with the app.

The comprehension questions about the project were answered correctly by most of the participants (Table 8). Question 2 had the fewest correct answers, with some people ticking two options, one of which would have been correct. Seven people did not answer the last question (open question).

## 5. Discussion

AAL systems are designed to help older people manage their everyday lives. Training is usually required for these systems to function in everyday life [6,7,8]. Against the background of demographic development with decreasing human resources, the support of training in new technologies by coaching robots could be helpful especially for younger seniors (the baby boomer generation).

With the development of a robo-coach, we have taken a step towards training by a social robot. The robo-coach and its three functions were tested in a study in Austria with 34 participants. Although a large part of the population is familiar with digital tools such as smartphones, tablets and the Internet, the majority of them have never had the opportunity to interact with a robot. This fact can have a direct impact on the perception of this technology and lead to acceptance problems. Therefore, in this study, we investigated participants’ expectations and perceptions before and after a coaching session with a robot. To determine whether the training worked, comprehension questions and tasks were asked afterwards.

The robot had a face, tactile abilities and could speak. Coaching by the robot was possible via three functions (i) Project Introduction, (ii) System Introduction and (iii) Fill-in Questionnaire. The AgeWell project and the Embodied Conversational Agent developed there served as the first application example. The following contributions to the literature are hereby made.

Although the participants were familiar with common digital tools, they showed scepticism about using the robot before interacting with it, which was particularly evident in the ATI scale and its sub-items. Evaluation of their expectations, prior to any interaction with the robot, showed that they overestimated the complexity of the robot and its functions and underestimated their ability to operate the robot without assistance. This finding demonstrates the importance of using real robots when conducting studies [17,47]. More specifically, our study is one of the few in the literature to report findings on older adults’ perceptions of a robot as a result of using a real robot. This result was homogeneous between tech-savvy and non-tech-savvy participants. Non-tech-savvy participants were less curious about testing new systems. This aspect could affect the future adoption of this technology and hinder its implementation in real-world environments. This finding underscores the need to provide coaching with real robots to older adults, even those familiar with the technology, to help them overcome some unrealistic assumptions that may be preventing them from fully using the technology. Interestingly, participants’ expectations changed noticeably after they used the robot. Contrary to their expectations, participants found that the robot was easy to use and that they did not need help to operate it. The majority showed a neutral to very positive attitude toward the robot’s functions, as measured by the AttrakDiff questionnaire. They also perceived the three functions and the interaction with the robot as very smooth. Although these results confirm evidence from related studies (e.g., [48]), they should be interpreted with caution. Participants’ pre-use expectations could not be influenced by design factors as they were measured before interacting with the system. Post-use perceptions, on the other hand, are strongly influenced by several factors such as the robot itself, the UX/UI design, and the participants’ white-collar nature. Thus, generalizing these interpretations of the results remains subject to further research. However, they suggest that policymakers may focus on familiarizing older adults with less common digital tools as a way to overcome negative expectations they may develop towards these technologies.

The questions that were used to check whether the participants had understood the content of the training could largely be answered. Part of the tasks concerning the ‘System Introduction’ could also be solved well. The participants had difficulties with one task in particular. Since the ‘System Introduction’ function was rated worse by the participants than the others, we conclude that the usability problem of the ECA already became apparent during the use of the robot. The users were then unable to distinguish between the robot and the ECA and therefore rated the function worse.

One limitation of this study that may have affected the results is the small number of participants. In addition, although the study used a real robot, it was conducted in a laboratory setting. In future work, the study could be extended by using the robot in real households over a longer period of time. In this way, the actual usefulness of a robo-coach as well as its perception by users in a real environment could be evaluated. In addition, other use cases for coaching should be tested, including assistance with everyday tasks.

## 6. Conclusions

The study showed that even younger seniors have reservations about new technologies in some cases. Moreover, many misjudge the complexity and ease of learning before using such technologies. However, this quickly subsided after the first interaction with the robot. After use, they showed a consistently positive attitude towards the robot. However, this also shows how important it is for (older) people to have the opportunity to try out new technologies in order to reduce their fears.

## Figures and Tables

**Table 1:** Surveys and measures.

	Data Collection	Measures
**Demographic survey**	Accompanist	Demographic data Age Gender Place of residence and postcode Highest educational attainment Employment status Years in/to retirement
**Pre-intervention survey**	Participants	Affinity for technology Use of smartphone or tablet Affinity for technology interaction (ATI) scale Expectations of the participants Two selected and adapted questions of the System Usability Scale (SUS) User Experience Questionnaire UEQ short version
**Post-intervention survey**	Participants	Perceptions of the participants System Usability Scale (SUS) User Experience Questionnaire UEQ short version AttrakDiff (Adapted)
**Comprehension tasks/questions**	Participants	Project Introduction Three comprehension questions with given choices One open question
	Accompanist	System Introduction Nine tasks had to be performed with the ECA shown in the introduction

**Table 2:** Demographic profile of the participants (*n* = 34).

	Description	Number	Percentage
**Gender**	female	23	68%
	male	11	32%
**Age**	55–58	10	30%
	59–62	13	38%
	63–66	11	32%
**Highest completed education**	Compulsory school	2	6%
	Apprenticeship	4	12%
	Professional school	8	24%
	A-levels	6	17%
**Employment status**	University education	14	41%
	Employed	17	50%
	Retired	17	50%

**Table 3:** Smartphone and tablet usage (*n* = 34).

	Description	Number	Percentage
**Smartphone users**	female	23	100%
	male	11	100%
**Tablet users**	female	13	57%
	male	7	64%

**Table 4:** Summary of the ATI scale based on the educational level (*n* = 34).

	Mean	std	Min	Max	Total	Age (Mean)	Age (std)	>3.5	<3.5
Compulsory school, apprenticeship or professional school	3.46	1.13	2.22	5.67	14	59.64	2.46	6	8
A-levels, university education	3.12	0.93	1.56	5.44	20	61.65	3.45	5	15
Overall	3.26	1.00	1.56	5.67	34	60.82	3.20	23	11

**Table 5:** Results for UEQ-S (*n* = 34). * *p* values < 0.001.

		Before Using the Robot			After Using the Robot			T-Test				
Negative	Positive	Mean	Variance	Std.	Mean	Variance	Std.	Upper	Lower	t	df	Sig.
**Pragmatic Quality**		0.86			2.0							
obstructive	supportive	1.62	1.4	1.2	2.1	1.5	1.2	−1.02	0.14	−1.5	66	0.13
complicated	easy	0.26	2.0	1.4	2.2	1.3	1.1	−2.56	−1.32	−6.2	66	0.00 *
inefficient	efficient	0.82	1.1	1.0	1.4	2.1	1.5	−1.18	0.05	−1.8	65	0.07
confusing	clear	0.79	1.4	1.2	2.4	0.7	0.8	−2.06	−1.05	−6.1	66	0.00 *
**Hedonic Quality**		2.17			1.6							
boring	exciting	2.06	1.5	1.2	1.4	2.9	1.7	−0.06	1.36	1.8	66	0.07
not interesting	interesting	2.29	0.9	1.0	1.6	2.2	1.5	0.07	1.28	2.23	66	0.02
conventional	inventive	2.00	1.8	1.3	1.7	2.6	1.6	−0.39	1.04	0.89	66	0.37
usual	leading edge	2.32	1.3	1.1	1.8	2.7	1.6	−0.15	1.21	1.54	66	0.12
**Overall**		1.52										

**Table 6:** Summary of the SUS scale based on the educational level (*n* = 34).

	Mean	std	Min	Max	Total	Age (mean)	Age (std)	>3.5	<3.5
Compulsory school, apprenticeship or professional school	83.04	10.84	60.00	97.50	14	59.64	2.46	8	4
A-levels, university education	73.04	16.13	45.0	95.0	20	61.65	3.45	8	11
Overall	77.43	14.79	45.00	97.50	34	60.82	3.20	16	15

**Table 7:** Tasks to be completed (*n* = 34).

Task	Number of Successfully Performed Tasks	Percentage (%)
Open the AgeWell application	34	100
Navigate to the physical activity section	34	100
Add an activity for the upcoming week	26	76
Delete the activity you have just added	16	47
Go back to the dashboard of the physical activity	34	100
Open the AgeWell application	34	100
Navigate to the mental health section	33	97
Add an activity for the upcoming week	26	76
Complete the selected activity	30	88

**Table 8:** Comprehension tasks/questions (*n* = 34).

Questions	Number of Correct Answers	Percentage (%)
What are the components of the AgeWell System?	30	88
For which population group is the AgeWell system developed?	24	71
The term AAL means: …	32	94
What kind of support does the AgeWell system give?	27	79

## Data Availability

The data presented in this study are available on request from the corresponding author. The data are not publicly available due to privacy concerns.

## Abbreviations

The following abbreviations are used in this manuscript:

AAL	Ambient or Active Assisted Living
ATI	Affinity for technology
ECA	Embodied Conversational Agent
EDR	Elderly Dependency Ratio
SUS	System Usability Scale
UEQ-S	User Experience Questionnaire UEQ short version
